# Expression of miRNAs in the Relationship Between Periodontitis and Cardiovascular Diseases: A Systematic Review

**DOI:** 10.3390/ncrna11030037

**Published:** 2025-05-06

**Authors:** Montiel Guerrero-Sabater, María Cosín-Villanueva, Pedro Almiñana-Pastor, Andrés López-Roldán

**Affiliations:** Department of Stomatology, Faculty of Medicine and Odontology, University of Valencia, 46010 Valencia, Spain; monguesa@alumni.uv.es (M.G.-S.); mariacosin27@gmail.com (M.C.-V.); andres.lopez-roldan@uv.es (A.L.-R.)

**Keywords:** cardiovascular disease, epigenetics, microRNA, periodontitis

## Abstract

Objectives: Periodontitis is a chronic inflammatory disease that could influence the pathophysiology of cardiovascular diseases through immunoinflammatory and epigenetic mechanisms. MicroRNAs (miRNAs) could be key mediators in this interaction, regulating gene expression and the synthesis of inflammatory molecules. The objective of this systematic review was to evaluate the relationship between periodontitis and cardiovascular diseases by analyzing the expression of miRNAs involved in both pathologies. Methods: A systematic search was performed in the PubMed, Scopus, Embase, and Web of Science databases following the PRISMA guidelines. A total of 320 studies were identified, of which seven were included after applying eligibility criteria. Data on study design, sample characteristics, periodontal and cardiovascular diagnostic methodology, and the analyzed miRNAs were extracted. Results: The included studies were observational case-control studies in humans (*n* = 5) and experimental studies in animal models (*n* = 3). The miRNAs selected by the studies to link both pathologies were miR-155, miR-155-5p, miR-146a, miR-143, miR-145, and miR-23b. Most studies observed the overexpression of these miRNAs in patients with periodontitis and cardiovascular disease, with miR-146a being the most frequently associated. Conclusions: The findings suggest that certain miRNAs, particularly miR-146a, may play a key role in the connection between periodontitis and cardiovascular disease. Its overexpression in patients with both pathologies reinforces the hypothesis of its involvement in the inflammatory processes associated with both conditions. It would be interesting to conduct studies to validate their clinical applicability as biomarkers of susceptibility to cardiovascular disease.

## 1. Introduction

Periodontitis is a chronic, inflammatory, and multifactorial disease whose primary etiological factor is bacterial plaque. Its progression is significantly influenced by host risk factors. Clinically, periodontitis presents periodontal pockets, gingival bleeding, and destruction of the tooth-supporting apparatus or periodontium, which consists of the periodontal ligament, connective tissue attachment, and alveolar bone. Consequently, this leads to clinical attachment loss, measured through the clinical parameter probing depth [1]. Persistent or chronic periodontal inflammation results in sustained secretion of proinflammatory mediators, causing tissue damage, bone resorption, and disease progression. This inflammation creates an altered environment both locally and systemically, thereby exacerbating systemic conditions such as diabetes [2] and cardiovascular diseases [3]. The association between periodontitis and various systemic diseases such as diabetes, obesity, Alzheimer’s, and cardiovascular diseases has been extensively studied in recent years [2,3].

Cardiovascular diseases encompass various cardiac and vascular conditions, including ischemia, atherosclerosis, peripheral arterial disease, infectious endocarditis, and acute myocardial infarction. Among these, atherosclerosis is a condition that can lead to both ischemia and acute myocardial infarction due to blood flow obstruction [4,5].

The pathophysiological pathways involved in the interaction between periodontal pathogens and atheroma plaque formation are related to the effects of bacteria on platelets, autoimmune responses (antibodies), and the invasion and/or absorption of bacteria (*Porphyromonas gingivalis* via its *fimbriae*) into endothelial cells and macrophages [6,7,8].

The role of genetics in the predisposition to both diseases has not been clearly established according to the current research. Genetics has failed to identify individuals susceptible to developing both comorbidities, likely due to the static nature of genetic markers. Therefore, in these chronic inflammatory diseases, epigenetic markers may represent a turning point in understanding their etiopathogenesis and susceptibility. These epigenetic markers are not static and, thus, can define changes in the cellular environment. In these chronic inflammatory conditions, the cellular microenvironment is exposed to various environmental stimuli that significantly influence the host’s immunoinflammatory response [9].

Although numerous studies have attempted to identify specific genes involved in the immunoinflammatory mechanisms linking periodontitis and cardiovascular diseases, the results have been inconsistent and inconclusive. In this context, epigenetics—and more specifically, miRNAs—could provide a key approach to understanding how environmental and molecular factors modulate gene expression without altering DNA sequences, thereby mediating the regulation of inflammatory processes [10,11,12].

The aim of this study was to analyze the relationship between periodontitis and cardiovascular diseases and to identify the miRNAs involved in the association between both conditions.

## 2. Results

### 2.1. Study Selection and PRISMA Flow Diagram

Following the application of the search strategy across different databases, a total of 75 records were identified in Embase, 44 in PubMed, 87 in Scopus, and 114 in Web of Science. After removing duplicates (*n* = 144), 176 studies remained for title and abstract screening. At this stage, 134 studies were excluded, primarily because they were narrative reviews or did not align with the PECO framework of this systematic review.

A total of 42 studies met the inclusion criteria. After full-text assessment, 34 studies were excluded for the following reasons: investigation of different genetic mechanisms (*n* = 7) or unrelated diseases (*n* = 4), absence of a control group (*n* = 6), publication in languages other than English or Spanish (*n* = 5), or classification as systematic reviews (*n* = 12).

**Figure 1:**
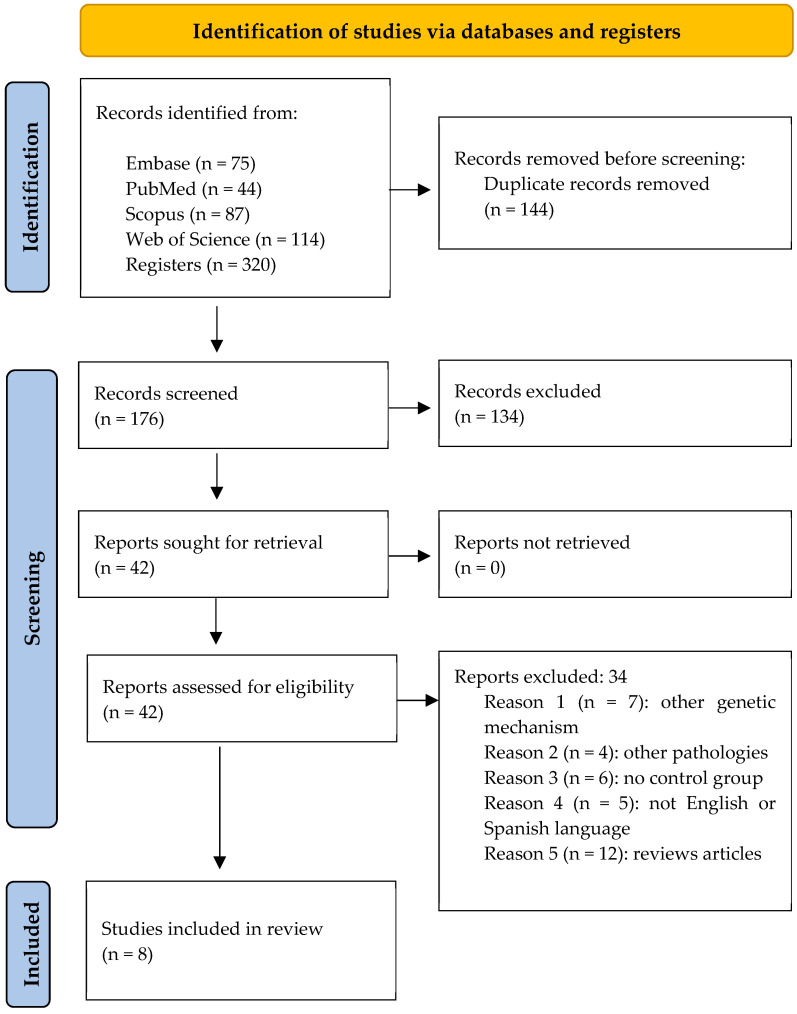
PRISMA flow diagram of the search process across the different databases.

Ultimately, eight studies were included in the qualitative synthesis. The PRISMA flow diagram illustrates the article selection process (Figure 1).

### 2.2. Qualitative Synthesis of Included Studies

Table 1 illustrates the extracted data from human studies. All of them were observational case-control studies. Sample sizes ranged from 37 [13] to 264 [14].

In all human studies, miRNA expression was compared between patients with both periodontitis and cardiovascular disease and healthy controls for both conditions. Additionally, the studies by Mahendra et al. (2021) [15] and Yagnik et al. (2019) described the cardiovascular parameters assessed [16].

For periodontal diagnosis, three out of the four studies used the 1999 Armitage classification, while one study applied the current 2018 Classification of Periodontal and Peri-Implant Diseases and Conditions [17].

On the other hand, Table 2 summarizes the data from animal experimental studies. In this case, sample sizes ranged from 16 [18] to 25 [19]. All selected animals were male ApoE−/− mice. Moreover, in one of the studies, a gingival biopsy was performed [20].

The miRNAs analyzed in the included studies were as follows:

- miR-155
- miR-155-5p
- miRNA-146a
- miR-143
- miR-145
- miR-23b
- miR-126

According to the included studies, most of the analyzed miRNAs were overexpressed in patients with periodontitis or in mice infected with periodontal pathogens and presenting cardiovascular disease, with miR-146a being the most frequently investigated [14,15,16,18,19]. However, only one of them reported low levels of miR-126 in patients with both cardiovascular and periodontal disease [15].

### 2.3. Quality Assessment

A risk of bias analysis was conducted for the observational human studies (Table 3) using the Newcastle–Ottawa Scale (NOS). Overall, the quality assessment indicated high-quality studies, as most received ≥7 stars, suggesting a low risk of bias.

Additionally, Table 4 presents the quality assessment for in vitro and animal studies using the SYRCLE’s Risk of Bias Tool. The three evaluated studies exhibited a moderate to high risk of bias, primarily due to insufficient information on randomization, blinding, and sample selection—key factors in ensuring internal validity in animal model studies [21].

**Table 1:** Characteristics and main results of included human studies.

Author and Year	Country	Sample Size	Type of Study	Periodontal Stage and Grade	Studied miRNAs	Other Studied Markers	Type of Sample	Periodontal Parameter Results	miRNA Expression Results
Zina A. Daily et al. (2023) [22]	Irak	*N* = 120 G1 (C): *n* = 30 G2 (P without AS): *n* = 30: G3 (AS without P): *n* = 30 G4 (AS + P): *n* = 30	Case-control	Stages III and IV Grades B and C	miR-155	IL-1b	Blood analysis	IL-1β higher in G2, G3, and G4 compared to G1	Upregulation of miR-155 in G2, G3 and G4 compared to G1
Wen-Wen Yang et al. (2024) [13]	China	*N* = 37 PLASMA SAMPLES FOR SEQUENCING ANALYSIS: *n* = 12: -G1(CP without CAS): *n* = 6 -G2 (CP + CAS): *n* = 6 PCR ANALYSIS (PLASMA): *n* = 25): - G3 (low/moderate CP without CAS): *n* = 6 - G4 (severe CP): *n* = 6 - G5 (low/moderate CP + CAS): *n* = 6 - G6 (severe CP + CAS): *n* = 7	Case-control	Chronic periodontitis	miR-155-5p		Plasma samples		Upregulation of miR-155-5p in CP + CAS compared to CP alone miR-155-5p overexpressed in plasma exosomes of patients with carotid atherosclerosis (CAS) + chronic periodontitis (CP) and in CAS tissue samples
Krutika Yagnik et al. (2019) [16]	India	*N* = 90 G1 (CP + CDH): *n* = 30 G2 (CP without CDH): *n* = 30 G3 (HP): *n* = 30	Case-control	Chronic periodontitis	miR-146a	PI, BOP, PPD, CAL Cardiovascular parameters: cholesterol, high- and low-density lipoproteins, triglycerides, systolic and diastolic blood pressure	Subgingival plaque samples collected with Gracey curettes (Hu-Friedy)		Upregulation of miRNA-146a and elevated cardiovascular and periodontal parameters in G1 compared to G2 and G3 Higher parameters in G2 compared to G3
J. Bagavad Gita et al. (2018) [14]	-	*N* = 264 G1 (ACS without CP): *n* = 66 G2 (ACS + CP): *n* = 66 G3 (CP without ACS): *n* = 66 G4 (HP): *n* = 66	Case-control	Moderate and severe chronic periodontitis	miR-146a	TNF-a, IL-1β, IL-6	Blood analysis and subgingival plaque samples collected with a sterile paper point	G1 overexpression (*p* < 0.001) compared to G3 and G4 G4 showed a minimal level of cytokine expression	Upregulation of miR-146a in of G2 miR-146a expressions were higher (G1, G2, and G3) compared to G4
Mahendra et al. (2021) [15]	India	*N* = 75 G1 (GP + CAD) *n* = 25. - G1a (*n* = 25): subgingival plaques samples - G1b (*n* = 25): coronary plaque samples at the time of CABG - G2 (GP) *n* = 25 - G3 (HP) *n* = 25	Case-control	Chronic periodontitis	miR-146a and miR-126	PI, BOP, PPD, CAL Cardiovascular parameters: SP, DP, HDL-c, LDL-c, TG, TC	Subgingival plaque samples collected with Gracey’s curette Coronary plaque samples were obtained at the time of CABG		Upregulation of miR-146a and downregulation of miR-126 in G1

G: group, CABG: coronary, artery bypass grafting surgery, miR: microRNA, HP: periodontally healthy, C: control, AS: aterosclerosis, P: periodontitis, CP: chronic periodontitis, CAS: carotid aterosclerosis, CDH: coronary heart disease, IP: plaque index, BOP: bleeding on probing, PPD: periodontal probing depth, CAL: clinical attachment level, TNF-a tumor necrosis factor-alpha, IL-1β: interleukin-1β, IL-6: interleukin-6, SP: systolic blood pressure, DP: diastolic blood pressure, HDL-c: high-density lipoprotein levels, LDL-c: low-density lipoprotein levels, TG: total triglyceride levels, TC: total cholesterol levels, *P. gingivalis*: *Porphyromona gingivalis*.

**Table 2:** Characteristics and main results of included experimental studies.

Author and Year	Country	Sample Size	Type of Study	Type of Sample	Studied miRNAs	Other Studied Markers	miRNA Expression Results	Conclusions
Jieyu Zhou et al. (2022) [18]	China	*N* = 16 (6-week-old male ApoE −/− mice) G1 (infected group): *n* = 8 Oral inoculation of *Fusobacterium nucleatum* in 100 μL of 4% CMC-PBS G2 (control): *n* = 8 100 μL of 4% CMC-PBS without bacteria (sterile vehicle)	Experimental	-	miR-146a, miR-23b and miR-155	IL-6, IL-1β, TNF-a, MCP-1, PCR, ox-LDL	miR-146a, miR-155, and miR-23b showed elevated levels in serum and aortic tissues of infected mice, although miR-155 expression in the aorta was not significantly higher	Serum levels of pro-atherosclerotic factors and microRNAs (miR-146a, miR-155, miR-23b) significantly increased after *F. nucleatum* stimulation, while HDL-c levels decreased
Md A. Nahid et al. (2011) [19]	-	*N* = 25 (male ApoE −/− mice) G1 (infected group): *n* = 15: G2 (control): *n* = 10	Experimental	-	miR-146a	TNF-a, IL-1β	Upregulation of miR-146a in spleens of mice infected with periodontal pathogens compared to the spleens of controls Upregulation of miR-146a in the maxilla of mice exposed to *P. gingivalis*, *T. denticola*, and *T. forsythia*	-
Hanyu Xie et al. (2023) [20]	China	*N* = 18 G1 (control mice ApoE−/− infected with *P. gingivalis*): *n* = 6 G2 (miR-143/145-deficient mice group: miR-143/145−/−ApoE−/−): *n* = 6 G3 (miR-143/145 overexpression mice group in LysM lineage cells (ApoE−/−LysMcre + rAAV9-miR-143/145): *n* = 6	Experimental	Gingival biopsy	miR-143/145	-	Upregulation of miR-143/145 in G3: Atherosclerotic lesions, osteoclasts, and apoptotic cells were significantly increased compared to G1	-

G: group, CMC-PBS: carboxymethyl cellulose, miR: microRNA, TNF-a tumor necrosis factor-alpha, IL-1β: interleukin-1β, IL-6: interleukin-6, HDL-c: high-density lipoprotein levels, MCP-1: monocyte chemoattractant protein-1, PCR: polymerase chain reaction, ox-LDL: oxidized low-density lipoprotein, ApoE: apolipoprotein E knockout, LysM: lysosome M, *P. gingivalis*: *Porphyromona gingivalis*, rAAV9: recombinant adeno-associated virus serotype 9.

**Table 3:** Quality assessment of observational studies based on the Newcastle–Ottawa Quality Assessment Scale (NOS).

		Selection			Comparability		Exposure		
Case-Control Study	Case Definition *	Representativeness of the Cases *	Selection of Controls *	Definition of Controls *	The Groups Are Comparable, and the Most Important Confounding Factor Is Controlled *, as Well as Other Factors **	Ascertainment of Exposure *	Same Method of Ascertainment for Cases and Controls *	Non-Response Rate *	Total
Zina A. Daily et al. (2023) [22]	*	*	*	*	*	*	*	*	8/9
Wen-Wen Yang et al. (2024) [13]	*	*			*	*	*		5/9
Krutika Yagnik et al. (2019) [16]	*	*	*	*	**	*	*	*	9/9
J. Bagavad Gita et al. (2018) [14]	*	*	*	*	*	*	*		7/9
Mahendra et al. (2021) [15]	*	*	*	*	*	*	*	*	8/9

**Table 4:** Quality assessment of experimental studies based on the SYRCLE’s Risk of Bias Tool.

Domain	Key Question	Jieyu Zhou et al. (2022) [18]	Md A. Nahid et al. (2011) [19]	Hanyu Xie et al. (2023) [20]
Animal Selection	Was the allocation sequence adequately generated and applied?	Unclear	Unclear	Unclear
Baseline Characteristics	Were the groups similar at baseline, or were confounding factors adjusted for in the analysis?	Unclear	Unclear	Unclear
Allocation Concealment	Was the allocation to different groups adequately concealed?	Unclear	Unclear	Unclear
Investigator Blinding	Were the animals randomly housed during the experiment?	No	Unclear	Unclear
Outcome Assessor Blinding	Were the caregivers and/or investigators blinded to the intervention each animal received during the experiment?	Unclear	No	No
Incomplete Data	Were animals randomly selected for outcome assessment?	Unclear	Unclear	Unclear
Selective Reporting	Was the outcome assessor blinded?	Unclear	Unclear	Unclear
Housing Conditions	Were incomplete outcome data adequately addressed?	Yes	Yes	Yes
Funding Sources and Conflicts of Interest	Were the study reports free from selective outcome reporting?	Unclear	Unclear	Unclear
Other Biases	Was the study apparently free from other issues that could result in a high risk of bias?	Unclear	Unclear	Unclear

## 3. Discussion

MicroRNAs (miRNAs) are small non-coding RNA molecules that regulate gene expression through post-transcriptional modulation of messenger RNA (mRNA). By participating in the regulation of various cellular processes, including inflammation, immunity, and disease development, their alteration has been linked to multiple pathologies, including periodontitis. Furthermore, their detection in biological fluids such as saliva, gingival crevicular fluid (GCF), and plasma makes them ideal biomarkers for the diagnosis and treatment of chronic inflammatory diseases, such as periodontitis. Therefore, this review was conducted to identify and analyze miRNAs simultaneously involved in both periodontitis and cardiovascular disease.

The sample size in observational human studies by Wen-Wen Yang et al. (2024) (*N* = 37) [13] and Krutika Yagnik et al. (2019) (*N* = 90) [16] was limited. Similarly, experimental animal studies by Jie-yu Zhou et al. (2022) (*N* = 16) [18], Md A. Nahid et al. (2011) (*N* = 25) [19], and Hanyu Xie et al. (2023) (*N* = 24) [20] also had small sample sizes. A small sample size may affect the validity of the results and compromise their reproducibility, limiting the generalization of conclusions.

Regarding diagnostic methods for sample collection, blood and plasma sample analyses were the most common. Only Krutika Yagnik et al. (2019) [16] analyzed subgingival plaque, while Hanyu Xie et al. (2023) [20] was the only experimental study that performed gingival biopsies. Biopsy is a more invasive procedure, diminishing the value of these miRNAs as biomarkers. GCF is a transudate derived from plasma that is localized in the periodontium. Additionally, it is directly related to periodontal inflammation, increasing in volume and undergoing qualitative molecular changes in the presence of pathology. Analyzing these miRNAs in GCF and assessing their potential to reflect systemic pathologies could be a promising avenue for further research [23].

Among the miRNAs studied in this systematic review, miRNA-146a stood out as a key regulator of inflammation by modulating the immune response through inhibition of the NF-κB pathway, a transcription factor directly involved in the activation of pro-inflammatory cytokines such as TNF-α, IL-1β, and IL-6, among others [24]. However, its role in periodontitis remains under investigation due to the complexity of the molecular interactions involved in this pathology.

J. Bagavad Gita et al. (2018) [14] analyzed the regulatory role of miR-146a in the immune-inflammatory response in patients with periodontitis and acute coronary syndrome. This microRNA was overexpressed in all patient groups. However, despite its negative regulatory effect on NF-κB, an increase in IL-1β and TNF-α expression was also observed, suggesting that its inhibitory action is insufficient to effectively reduce inflammation and that additional mechanisms and inflammatory molecules likely mediate this regulatory process.

This apparent contradiction can be explained by the complexity of the inflammatory signaling network. NF-κB activation is not solely dependent on miR-146a but can be induced by multiple stimuli, such as bacterial lipopolysaccharides (LPS), Toll-like receptors (TLR2 and TLR4), and other pro-inflammatory signaling pathways [25]. This means that while miR-146a exerts an inhibitory effect on NF-κB, other pathways may still promote its activation, maintaining inflammation in chronic periodontitis. In this context, inflammation is not a linear process but a network of dynamic interactions where multiple mediators regulate and modulate the immune response. This molecular interconnection makes it challenging to isolate the absolute effect of a single factor, which may explain why miR-146a overexpression does not result in a significant decrease in NF-κB and its pro-inflammatory cytokines, as observed in all biomolecular studies [26]. These findings confirm that periodontitis is a multifactorial disease, where inflammation regulation depends on a complex balance between multiple molecules and signaling pathways.

Systemic inflammation, triggered by obesity and amplified by periodontitis, constitutes a crucial link connecting these two disorders with the development of cardiovascular diseases. In this regard, Krutika Yagnik et al. (2019) [16] demonstrated a statistically significant correlation between periodontal and cardiac variables, BMI, and miRNA-146a levels in patients with both conditions [27].

The findings from animal models provide valuable insight into the actual impact of miR-143/145 on atherosclerosis accelerated by *P. gingivalis.* Modulation of this miRNA in mice directly influences disease progression, macrophage efferocytosis, and its interaction with Siglec-G, enhancing the understanding of underlying pathogenesis mechanisms. These results not only validate observations from in vitro studies but also open potential therapeutic approaches for human intervention [20].

Another studied microRNA, miR-155, is involved in several biological processes, including cell proliferation, immunity, and inflammation, and plays a role in inflammatory reactions and autoimmune diseases [28,29]. Zina A. Daily et al. (2023) [22] showed that patients with concurrent cardiovascular disease and periodontitis exhibited miR-155 expression levels 14.08 times higher in the blood compared to the control group.

In line with these findings, recent research has demonstrated that miR-155-5p-enriched exosomes derived from periodontal endothelial cells may play a key role in the progression of atherosclerosis. miR-155-5p has been found to be highly expressed in the plasma exosomes of patients with carotid atherosclerosis (CAS) and chronic periodontitis (CP), suggesting a cellular communication mechanism between these two pathologies. Additionally, bacterial products from periodontitis, such as lipopolysaccharide (LPS), have been shown to increase miR-155-5p expression, facilitating its transfer to vascular endothelial cells and promoting endothelial barrier disruption and angiogenesis. These results reinforce the hypothesis that periodontitis contributes to cardiovascular disease development through miR-155-mediated mechanisms [13,30].

Jieyu Zhou et al. (2022) [18] analyzed the pathogenicity of Fusobacterium nucleatum and observed that its infection was associated with increased periodontal parameters, the progression of atherosclerotic lesions, and an elevated expression of miR-146a, miR-155, and miR-23b. This microRNA is upregulated in individuals with cardiovascular diseases and found at lower levels in healthy subjects, as it plays a key role in cardiac development [31]. A similar study by Di et al. [32] aimed to investigate the role of this miRNA by analyzing circulating endothelial progenitor cells (EPCs), their neovascularization capacity, and their relationship with cardiovascular disease. The results showed that the inhibition of miR-23b promotes blood flow recovery in ischemic limbs in mice.

Among the miRNAs analyzed, miRNA-126 was the only one not found to be upregulated in patients with concomitant cardiovascular and periodontal disease [15]. This microRNA plays a key role in maintaining vascular homeostasis and modulating the inflammatory response, primarily through the negative regulation of pro-inflammatory cytokines and biomarkers that contribute to the amplification of inflammation in conditions such as atherosclerosis and diabetes. Its downregulation in affected individuals may reflect an impaired anti-inflammatory regulatory mechanism, thereby contributing to the progression of vascular and inflammatory damage associated with these comorbidities [33].

Other studies have found that microRNAs associated with other systemic inflammatory conditions, such as type 2 diabetes mellitus and obesity, include miR-146a, miR-155, miR-200b, miR-223, and miR-203 [34].

Previous studies, such as those by Radovic et al. (2018) [35] and Saito et al. (2017) [36], have reported elevated microRNA levels in the gingival crevicular fluid of periodontitis patients compared to healthy individuals. Specifically, Radovic et al. (2018) [35] evaluated miR-146a and miR-155 in patients with chronic periodontitis—with and without type 2 diabetes mellitus—observing a significant reduction in their expression after non-surgical periodontal treatment. This supports the hypothesis that miR-146a and miR-155 play a crucial role in periodontal inflammation regulation via TLR activation. Similarly, Almiñana et al. (2023) [9], after conducting a comprehensive screening of all existing miRNAs, identified miR-199b-3p and miR-146a as the most potent biomarkers in a sample of chronic periodontitis patients.

Further research with well-designed studies could enhance the understanding of these biomarkers and their role in susceptibility to periodontitis and cardiovascular disease, contributing to the development of precision medicine.

## 4. Materials and Methods

This systematic review was conducted following the PRISMA 2020 (Preferred Reporting Items for Systematic Reviews and Meta-Analyses) criteria [37]. The protocol was registered in PROSPERO under ID CRD420251023111.

- Review question.

The formulated Population, Exposure, Comparison, and Outcome (PECO) [38] question was as follows: Population (humans with myocardial ischemia and/or occlusive arterial disease), Exposure (periodontitis), Comparison (subjects with cardiovascular disorders but without periodontitis), and Outcome (miRNA expression levels). Therefore, the research question was “Do individuals with myocardial ischemia and/or occlusive arterial disease along with periodontitis exhibit greater alterations in miRNA expression levels compared to those with only cardiovascular disorders?”

- Inclusion and Exclusion Criteria.

Cohort studies, case-control studies, randomized controlled trials (RCTs), experimental studies using in vitro samples, and experimental studies on animals were included. Conversely, studies without a control group, case reports, systematic or literature reviews, and expert opinions were excluded.

- Search Strategy.

To formulate the search strategy, an initial terminological analysis was conducted. The process of formulating the search equations and their application in each of the databases was carried out by a single researcher (M.G.-S).

The search equation used was as follows: (MicroRNAs OR MicroRNA OR miRNA OR miRNAs OR “miRNAs” OR “Micro RNA” OR “Micro RNAs” OR “mi-RNA” OR “mi-RNAs” OR “mRNA” OR “mRNAs”) AND (“Periodontal disease” OR “Periodontal diseases” OR Periodontitis OR gingivitis OR Gingival OR Periodontal OR paradontoses OR paradontitis OR Chronic periodontitis) AND (“Myocardial Ischemia” OR “Ischemic Heart Diseases” OR “Ischemic Heart Disease” OR “Myocardial Ischemias” OR “Arterial Occlusive Diseases” OR “Arterial Occlusive Disease” OR “Arterial Obstructive Diseases” OR “Arterial Obstructive Disease” OR “Arterial Occlusion” OR “Arterial Occlusions” OR “Atherosclerosis” OR “Atheroscleroses” OR “Carotid artery diseases” OR “Carotid atherosclerosis” OR “Atherosclerotic plaque” OR “Atheroma” OR “Atheromas” OR “Atheromatous plaque” OR “Angina pectoris” OR “Acute coronary syndrome” OR “Peripheral arterial disease”).

To identify all studies addressing the PECO question, searches were conducted in the PubMed, Embase, Scopus, and Web of Science databases. No language or publication date filters were applied to prevent the loss of potentially relevant articles. The search process began on 31 December 2024 and concluded in January 2025.

The selection process was carried out by two reviewers (M.G.-S. and M.C.-V.), who evaluated the titles and abstracts of the articles available in the databases. To assess the level of agreement between the two reviewers, the inter-examiner agreement was measured using the Kappa coefficient. Additionally, duplicate articles were identified and removed. In cases of discrepancies, a third reviewer (P.A.-P.) was consulted.

If the title and abstract did not provide sufficient information to determine an article’s eligibility, a full-text review was performed. After this initial phase, the selected articles underwent a complete review, and in cases of exclusion, the reasons for rejection were documented.

- Data Extraction and Variable Lists.

Two tables were designed to distinguish human studies from animal experimentation studies.

For both tables, including those for human studies and experimental studies, the following data were extracted: author and year, country, sample size, study type, studied miRNAs, other biomarkers analyzed, sample type, and miRNA expression results.

Additionally, in the table for human studies, variables related to periodontal stage and grade, as well as the results of periodontal parameters, were included. Conversely, in the table for experimental studies, an additional column was incorporated to summarize the conclusions of each study.

- Quality Assessment.

To assess the quality of the selected human studies, the Newcastle–Ottawa Quality Assessment Scale (NOS) was used. This scale includes eight criteria evaluating different aspects of the study methodology. Each study can receive one point per fulfilled criterion, except for the comparability section, which allows for up to two points, resulting in a maximum score of nine points.

Similarly, to evaluate the quality of animal or in vitro experimental studies, the SYRCLE’s Risk of Bias Tool was employed. In this tool, “Yes” indicates a low risk of bias, “No” indicates a high risk of bias, and “Unclear” signifies an uncertain risk of bias.

## 5. Conclusions

Within the limitations of this systematic review, the studies seem to indicate that miRNAs could be adequate biomarkers in the study of the interrelationship between cardiovascular disease and periodontitis. Studies should be designed to determine whether these markers in the GCF could indicate susceptibility to both periodontitis and cardiovascular disease and improve their value as biomarkers.

## Data Availability

We have no complementary data; all the information regarding the methodology of this systematic review is reflected in this paper.
